# Does Autism Affect Children’s Identification of Ownership and Defence of Ownership Rights?

**DOI:** 10.1007/s10803-021-04872-6

**Published:** 2021-01-25

**Authors:** Calum Hartley, Nina Harrison, John J. Shaw

**Affiliations:** 1 Department of Psychology, Lancaster University, Lancaster, LA1 4YF UK; 2 Present Address: Division of Psychology, School of Allied Health Sciences, De Montfort University, Leicester, LE1 9BH UK

**Keywords:** Autism spectrum disorder, Ownership identification, Ownership rights, Pronouns, Typical development

## Abstract

This study investigated how autism spectrum disorder (ASD) impacts children’s ability to identify ownership from linguistic cues (proper nouns vs. possessive pronouns) and their awareness of ownership rights. In comparison to typically developing (TD) children matched on receptive language (*M* age equivalents: 53–56 months), children with ASD were less accurate at tracking owner-object relationships based on possessive pronouns and were less accurate at identifying the property of third parties. We also found that children with ASD were less likely to defend their own and others’ ownership rights. We hypothesise that these results may be attributed to differences in representing the self and propose that ASD may be characterised by reduced concern for ownership and associated concepts.

In order to become effective navigators of the social world, children must learn to identify ownership and adhere to rules that regulate interactions with property (Brown 1991). Identifying ‘who owns what’ is complicated by the fact that ownership is not a physical attribute that can be visually perceived (Blake and Harris 2009; Friedman and Neary 2008). Rather, ownership is an invisible social construct that is usually inferred from heuristics (e.g. physical possession) or established through linguistic communication (Ross et al. 2015). Once ownership has been identified, humans adjust their behaviour according to ‘ownership rights’ that determine how we interact with self- and other-owned property. Typically developing (TD) children develop the concept of ownership through social interactions with others (Kanngiesser et al. 2015). However, learning of this nature can present a challenge for children with autism spectrum disorder (ASD) who experience difficulties associated with communication and interaction (APA 2013). The purpose of this study is to investigate how ASD impacts children’s ability to identify ownership from linguistic cues and their understanding of ownership rights.

The use of language to identify and establish ownership emerges early in typical development. TD children use first-person possessive pronouns (e.g. “mine”) to disambiguate objects in their environment from 12 months (Saylor et al. 2011) and produce first-person pronouns to denote their ownership of objects by 18 months (Fasig 2000; Hay 2006). By 24 months, TD children use second-person possessive pronouns when referring to objects (e.g. “yours”) and are capable of accurately identifying self- and other-owned property using proper nouns and possessive pronouns (Lewis and Ramsay 2004; Brownell et al. 2013). More accurate and frequent production of possessive pronouns is associated with more frequent engagement in physical altercations over toys and greater likelihood of sharing with others—behaviours that are indicative of ownership understanding (Brownell et al. 2013; Hay 2006). When engaging in social play, toddlers spontaneously provide ownership information for their peers. Ross et al. (2015) reported that, when interacting with toys, the most frequent comments made by children aged 24–30 months concerned ownership. Children often referenced ownership status when their toys were first introduced and when attempting to take their toys from friends. Thus, language plays a fundamental role in scaffolding children’s identification of ownership and enabling them to communicate their relationship to property such that others can behave in accord with ownership rights.

Ownership rights refer to cultural rules specifying that owners have the right to use and control access to their property. From 18 months, TD children engage in disputes over property access and protest against peers’ attempts to claim their toys (Bakeman and Brownlee 1982; Hay and Ross 1982). By 2 years, TD children explicitly refer to their ownership rights in disputes with siblings and peers (Eisenberg-Berg et al. 1979; Ross 1996, 2013), but they do not necessarily understand that others have ownership rights too (although see Ross et al. 2015). In Rossano et al. (2011), a puppet attempted to claim and dispose of objects belonging to TD two- and 3-year-old participants and an experimenter. While both age groups protested when their own property was under threat, they were significantly less likely to defend the experimenter’s ownership rights. However, it is possible that the relatively more frequent protests in defence of children’s property were due to pre-existing preferences for those objects (in contrast to the experimenter’s objects) rather than genuine understanding of ownership rights. This issue is addressed by Kanngiesser and Hood (2014a) who tested whether TD 2- and 3-year-olds would defend ownership rights when a puppet attempted to steal newly-created property (e.g. drawings) belonging to them and an experimenter. Both age groups claimed ownership of items they created and defended their own property rights via physical or verbal intervention. The 3-year-olds additionally attributed ownership to the experimenter, but rarely defended their rights.

To date, relatively little is known about the impact of ASD on children’s development of ownership understanding and only a single study (to our knowledge) has examined accuracy of ownership identification in this population. In Hartley and Fisher (2018a), TD children and children with ASD matched on receptive vocabulary were randomly assigned one of three toys to keep, before being offered the chance to trade for an alternative. The remaining objects were then allocated to the experimenter and a puppet. When participants were asked to match the objects to their respective owners, both populations demonstrated highly accurate and comparable tracking of owner-object relationships. However, it is important to note that ownership relationships were established with reference to each party’s name (e.g. “This toy is for Jack”) and ownership questions also referenced each party’s name (e.g. “Which toy belongs to Jack?”). Therefore, it is possible that participants’ responding in this study was strengthened by the explicit associations between objects and the names of their corresponding owners.

It is plausible that children with ASD may experience difficulty identifying ownership when relationships between people and property are established and probed using pronouns rather than proper nouns. Atypical pronoun reversals (e.g. saying “I” instead of “you”, and vice versa) are a well-documented feature of language and communication in children with ASD (Kanner 1943; Luyster and Lord 2009; Tager-Flusberg et al. 2005). While these kinds of errors can be observed in TD children (e.g. Evans and Demuth 2012), they are a more common characteristic of speech in autism (Dale and Crain-Thoreson 1993; Evans and Demuth 2012; Overweg et al. 2018; Tager-Flusberg 1994). Tager-Flusberg (1994) reported that 13% of personal pronouns produced by children with ASD were reversed (although for lower estimates, see Barokova and Tager-Flusberg 2019, and Naigles et al. 2016). Overweg et al. (2018) and Mizuno et al. (2011) observed differences in speech interpretation that resulted in pronoun comprehension errors in children and adults with ASD respectively. Studies have also found that children with ASD are more likely to use proper nouns to avoid using pronouns (Shield and Meier 2014).

Unlike proper nouns, which have a fixed referent, personal pronouns require deictic shifting—the speaker/listener must continuously remap the same pronoun to different people depending on who is speaking (Hartmann and Stork 1972; Levinson 1983). It has been proposed that personal pronouns are particularly challenging for children with ASD because difficulties representing the self in relation to others (e.g. Charney 1980; Hobson 1990, 1993; Overweg et al. 2018) and/or differences in executive functioning (e.g. Dale and Crain-Thoreson 1993) impact their ability to understand or shift between different speakers’ perspectives. The present study is the first to examine the accuracy of children with ASD when interpreting possessive pronouns, which also require deictic shifting.

No previous studies have investigated understanding of ownership rights in children with ASD. However, evidence that ASD influences important aspects of ownership-related cognition could signpost potential differences in understanding of ownership rights. In experiment 1 of Hartley and Fisher (2018a), TD children showed a clear preference for their randomly assigned toy and traded infrequently (demonstrating a “mere ownership effect”; Gelman et al. 2012; Harbaugh et al. 2001) while children with ASD often traded for a different object that they preferred. In subsequent experiments, children with ASD did not over-value self-selected toys in comparison to identical copies, or over-value randomly assigned toys in comparison to different other-owned toys or identical copies. These findings suggest that ownership-induced connections to *the self* do not irrationally bias how children with ASD evaluate objects, indicating the absence of an extremely robust cultural phenomenon that influences both the psychology of identity and economics (Belk 1988, 2000). Across two experiments in Hartley et al. (2020a), TD children perceived items belonging to famous owners (e.g. Winnie the Pooh’s honey jar) to be more valuable than similar items belonging to non-famous owners (e.g. my mum’s cookie jar). By contrast, children with ASD matched on receptive vocabulary did not over-value items with special ownership histories, but their valuations were moderated by object qualities unrelated to ownership (e.g. material value and newness). Together, the results of these studies suggest that children with ASD evaluate objects via an unusual strategy that prioritises material qualities over ownership history; they appear to be more concerned by what an object *is* rather than *whom* it is associated with.

Hartley et al. (2020a) propose that because ownership is a cultural convention (Kanngiesser et al. 2015; Sparks et al. 2016), decreased social motivation and social-cognitive difficulties that characterise ASD may reduce the frequency and quality of interactions through which children learn ownership norms (APA 2013; Chevallier et al. 2012). Furthermore, early differences in self-other understanding (Lind 2010) may reduce the psychological importance of property ownership to children with ASD (Hartley and Fisher 2018a; Hartley et al. 2020a). Children with ASD can have difficulty encoding and retrieving personally experienced events and information (e.g. Bruck et al. 2007; Goddard et al. 2007) and show reduced awareness of emotions and mental states (e.g. Ben Shalom et al. 2006; Hill et al. 2004; Silani et al. 2008; Williams and Happé 2010). Thus, for children with ASD, associating objects with the self and others may not elicit the myriad ownership-induced cognitive biases that are observed in TD children (Cunningham et al. 2013; Gelman et al. 2012; Kahneman et al. 1991). If children with ASD do not derive value from abstract relationships between people and objects, we may expect to observe diminished understanding and adherence to ownership rights as well.

The objective of this study was to examine ownership identification from linguistic cues and understanding of ownership rights in children with ASD. In one task, children were presented with sets of objects—one object belonged to the child and two objects belonged to other owners. In some trials, ownership relationships between objects and owners were stated and tested using proper nouns (e.g. “This is Nina’s lunch box.” and “Which lunch box is John’s”?). In other trials, ownership relationships were stated and tested using possessive pronouns (e.g. “This pencil case is yours” and “Which pencil case is mine?”). Based on prior evidence that children with ASD can have difficulty comprehending pronouns (e.g. Overweg et al. 2018), we predicted that they would be less accurate than TD children when identifying ownership based on possessive pronouns. However, we expected the groups to achieve similar accuracy when identifying ownership based on proper nouns (see Hartley and Fisher 2018a). In another task based on Kanngiesser and Hood (2014a), children created new objects (e.g. drawings) with two experimenters. One experimenter then attempted to claim ownership of all of the objects. Due to their reduced concern for ownership history (Hartley and Fisher 2018a; Hartley et al. 2020a), we anticipated that children with ASD would be less likely to protest in defence of their own and others’ ownership rights than TD children. Importantly, the results of this research will advance theoretical understanding of how ASD affects a crucial foundation of social-cultural cognition.

## Method

### Participants

Participants were 18 children with ASD (*M* age = 11.37 years, *SD* = 3.27, range 6.17–17.08 years) and 19 TD children (*M* age = 3.88 years, *SD* = 0.53, range 3.08–4.67 years) recruited from specialist schools, mainstream schools, and preschools. Samples were closely matched on receptive vocabulary as measured by the British Picture Vocabulary Scale (BPVS; Dunn et al. 1997; ASD *M* age equivalent = 4.64 years, *SD* = 1.49, range 2–6.83 years; TD *M* age equivalent = 4.39 years, *SD* = 1.06, range 2.83–6.08 years), *t*(35) = 0.60, *p* = .55. All children with ASD were diagnosed by a qualified educational or clinical psychologist using standardised instruments (e.g. Autism Diagnostic Observation Scale and Autism Diagnostic Interview-Revised; Lord et al. 1994, 2002) and expert judgement. Diagnoses were confirmed via the Childhood Autism Rating Scale 2 (CARS; Schopler et al. 2010), which was completed by each participant’s class teacher (ASD *M* score: 36.14; TD *M* score: 15.00). Children with ASD were significantly older than TD children *t*(35) = 9.85, *p* < .001, *d* = 3.20, and had significantly higher CARS scores, *t*(35) = 13.78, *p* < .001. All procedures performed in this research involving human participants were in accordance with the ethical standards of the institutional and national research committees. Informed consent was obtained from parents/caregivers prior to children’s participation.

### Materials

Stimuli for the ‘owner identification task’ included a variety of objects that belonged to the participants, plus different examples of the same objects that belonged to a male experimenter and a female experimenter. Six items belonging to each child were identified prior to testing, and the experimenters each sourced six items of the same type. While a minority of children with ASD required the use of unique items (e.g. small differently-coloured tins of Vaseline), the following items were common across many children: lunch box, shoe, hat, school bag, book, toy, jumper and drinking bottle.

Stimuli for the ‘ownership rights task’ included pens and paper, playdough, and a brightly-coloured box with removable lid.

### Procedure

Participants were tested individually in their own educational settings and were accompanied by a familiar adult. Children were reinforced throughout the session for attention and good behaviour, but did not receive feedback concerning their performance in the tasks.

#### Owner Identification Task

There were two within-subjects conditions delivered on different days: Naming and Pronoun. Half of the participants in each sample received the Naming condition in the first session, followed by the Pronoun condition in the second session approximately 1 week later. The other half of the participants in each sample experienced the two conditions in reverse order.

Children sat at a table opposite two adult experimenters (one male, one female). Each session began with the experimenters introducing themselves and inviting the child to play (e.g. Hello [child’s name], my name is John and this is my friend Nina. Let’s play a game!”). Children then completed three trials. In each trial, one experimenter presented one of the six pre-identified items belonging to the child (e.g. a blue lunch box), plus two other examples of the same type of item that belonged to the experimenters (e.g. a green lunchbox and a yellow lunchbox). Wherever possible, the experimenters’ items were gender neutral in terms of their colour and/or design, so owner-object matching could not be facilitated by awareness of social stereotypes. Children were allowed to explore the items for a few seconds before an experimenter placed each object in front of its owner and verbally stated the object-owner relations. These verbal statements differed between conditions. In the Naming condition, each owner was named explicitly (e.g. “This is [child]’s lunch box, it belongs to [child]. This is John’s lunch box, it belongs to John. This is Nina’s lunch box, it belongs to Nina.”). In the Pronoun condition, owners were referenced using possessive pronouns (e.g. “This is *your* lunch box, it belongs to *you*. This is *his* lunch box, it belongs to *him*. This is *my* lunch box, it belongs to *me*.”). The order that object-owner relations were highlighted was randomised across participants. An experimenter then placed the three items in the middle of the table (locations—left, middle, centre—were counterbalanced) and asked the participant to identify the owner of each object. These questions varied between conditions. In the Naming condition, the owners for each object were named (e.g. “Which lunch box is John’s? Which lunch box is Nina’s? Which lunch box is [child]’s?”). In the Pronoun condition, the owners were referred to using possessive pronouns (e.g. “Which is *yours*? Which is *hers*? Which is *mine*?”). The order of questions was counterbalanced across participants. Children responded by verbally or gesturally indicating which of the three items belonged to the stated owner. An experimenter then removed the objects from the table and initiated the next trial with different objects.

#### Ownership Rights Task

Children sat at a table opposite two adult experimenters (one male, one female). The craft-making materials were located in a box next to the table (neither experimenter indicated ownership of the materials at any time). Based on Kanngiesser and Hood (2014a), this task was delivered in a single session and involved two stages in a fixed sequence: 1. Warm-up, 2. Test stage.

##### Warm-Up

The session began with the experimenters introducing themselves and inviting the child to play (e.g. “Hello [child’s name], my name is John and this is Nina. Let’s play a game!”). Children then completed two warm-up trials to establish whether they would intervene if one of the experimenters behaved strangely. Experimenter 1 presented a small cup and stated their intention to “drink a cup of tea”. Experimenter 1 then pretended to drink from the cup upside down for about 30 s while stating “I’m drinking a cup of tea!”. If the child spontaneously corrected this odd behaviour, Experimenter 1 thanked the child and demonstrated the appropriate action. If the child did not spontaneously intervene, Experimenter 2 verbally encouraged the child to correct the unusual behaviour (e.g. “You should tell Nina if she is doing something wrong!”). Following the child’s intervention or the expiration of approximately 30 s, the second warm-up trial was administered. This followed an identical format, except Experimenter 1 pretended to brush their teeth using the handle of a toothbrush rather than the bristles.

##### Test Stage

Following the warm-up trials, Experimenter 1 placed a brightly-coloured box next to them on the table, stating that the contents of the box belonged to them (e.g. “This is Nina’s box. I always keep my things in this box. Everything in this box belongs to Nina!”). Children then completed two trials that involved drawing pictures or creating models from playdough (order counterbalanced across participants). At the start of each trial, Experimenter 2 presented the craft materials (playdough or paper and colouring pens) and suggested that all parties use them to create something (e.g. “Let’s all make/draw something! [Child] can make/draw something, John can make/draw something, and Nina can make/draw something!”). The child and experimenters then used the craft materials to draw a picture or make a model for approximately 1 min. The experimenters ensured that their creations were visually distinct from the child’s creation and from each other. Experimenter 2 then asked Experimenter 1 “what do you think of these drawings/models?”. Experimenter 1 pointed at one of the three drawings/models and stated “I really like this one! I want to keep it and put it in my box and never give it back.” After waiting for a few seconds, Experimenter 1 repeated their intention (“I love this one! I am going to take it and put it in my box and never give it back!”). Experimenter 1 then picked up the identified model/drawing and slowly moved to place it in their box. Experimenter 1 then repeated this behaviour until they had tried to claim all three of the newly-made objects. If the child protested against Experimenter 1’s attempt to claim an object, the object was left on the table. Verbal and nonverbal behaviours were categorised as a protest. Children could protest physically by retrieving an object from Experimenter 1 and possessing it for at least 10 s. Alternatively, children could protest verbally with or without explicitly referencing ownership (e.g. “no”, “don’t do that”, “I really like that one”, “you can’t have mine”, “that belongs to him”). After Experimenter 1 had attempted to claim each object, the three objects were placed in the middle of the table and Experimenter 2 asked the child to indicate the appropriate owner for each object (Ownership Questions; e.g. “Who should keep this? Point to who should keep this.”). We coded whether children attributed ownership to the person who made the object. After the participant had responded to these questions, the second trial was administered following the same format using the alternate crafting materials.

## Results

### Owner Identification Task

**Fig. 1:**
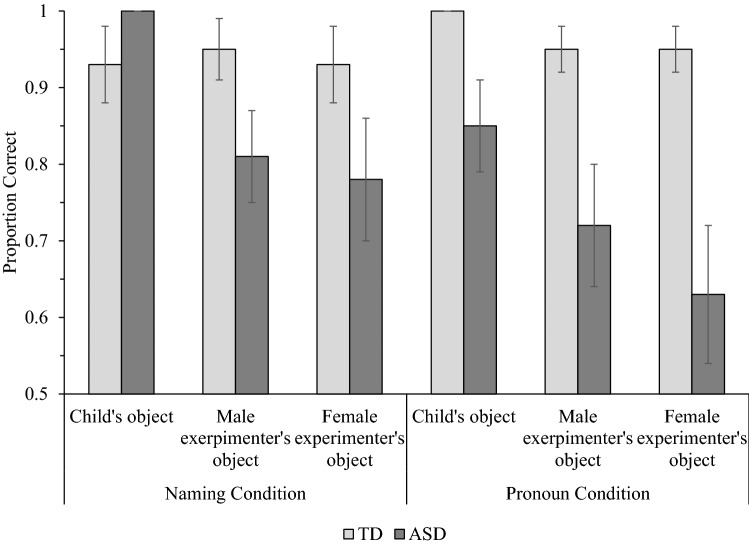
Mean accuracy for typically developing (TD) children and children with autism spectrum disorder (ASD) in the Naming and Pronoun conditions of the owner identification task. Error bars show ± 1 SE. All bars significantly exceeded chance (0.33) at *p* < .01

In both the Naming and Pronoun conditions, children were scored out of three on trials involving identification of their objects, identification of objects belonging to the male experimenter, and identification of objects belonging to the female experimenter (see Fig. 1). These data were entered into a 2(Population: TD, ASD) × 2(Condition: Naming, Pronoun) × 3(Owner: Child, Male Experimenter, Female Experimenter) mixed ANOVA.

Significant main effects of Owner, *F*(2, 70) = 9.61, MSE = 0.28, *p* < .001, η_p_^2^ = .22, and Population, *F*(1, 35) = 9.47, MSE = 1.20, *p* = .004, η_p_^2^ = .21, were qualified by a significant Population × Owner interaction, *F*(2, 70) = 6.00, MSE = 0.28, *p* = .004, η_p_^2^ = .15. Bonferroni-adjusted pairwise comparisons showed that TD children (*M* = 2.89) and children with ASD (*M* = 2.78) did not differ in accuracy when identifying objects belonging to them (*p* = .32). However, children with ASD were significantly less accurate than TD children when identifying items that belonged to the male experimenter (ASD *M* = 2.31; TD *M* = 2.84; *p* = .003) and the female experimenter (ASD *M* = 2.11; TD *M* = 2.82; *p* = .004). A repeated measures ANOVA revealed a significant effect of Owner for children with ASD, *F*(2, 34) = 8.53, MSE = 0.25, *p* = .001, η_p_^2^ = .33. Children with ASD identified objects belonging to them with significantly greater accuracy than items belonging to the male experimenter (*p* = .016) and the female experimenter (*p* = .008), which did not differ in accuracy (*p* = .70). There was no effect of Owner for TD children (*p* = .47), indicating no significant differences in accuracy when identifying items belonging to them, the male experimenter, and female experimenter.

The omnibus ANOVA also detected a borderline Population × Condition interaction, *F*(1, 35) = 3.93, MSE = 0.80, *p* = .055, η_p_^2^ = .10. Given our a priori hypotheses, we proceeded to deconstruct the relationship between Population and Condition, though note that the comparisons should be treated with caution as the interaction was marginally significant. Bonferroni-adjusted pairwise comparisons showed that the accuracy of children with ASD (*M* = 2.59) and TD children (*M* = 2.81) did not differ in the Naming condition (*p* = .24). However, TD children (*M* = 2.89) responded with significantly greater accuracy than children with ASD (*M* = 2.20) in the Pronoun condition (*p* = .002). While TD children did not differ in accuracy between the Naming and Pronoun conditions (*p* = .56), children with ASD tended to respond with greater accuracy in the Naming condition than the Pronoun condition (*p* = .058).

### Ownership Rights Task

Eighteen TD children corrected the experimenter’s unusual behaviour on at least one warm-up trial (94.74%) in comparison to 11 children with ASD (61%), a significant difference, χ^2^(1, N = 37) = 6.17, *p* = .013. These data suggest that that children with ASD were less likely to correct the experimenter’s erroneous behaviour, but they do not necessarily reflect children’s willingness to defend their own and others’ ownership rights. Indeed, corrective behaviour during the warm-up did not significantly correlate with protest behaviour in the subsequent test trials for TD children (all *R* values < .27, all *p* values > .27) or children with ASD (all *R* values < .29, all *p* values > .24).

**Fig. 2:**
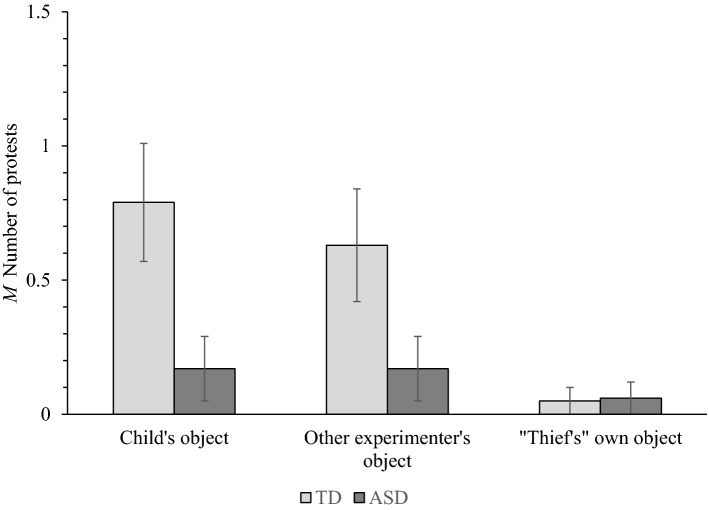
Mean number of protests made by typically developing (TD) children and children with autism spectrum disorder (ASD) in the ownership rights task. Error bars show ± 1 SE

Children were scored out of two corresponding to the number of trials on which they protested against an experimenter attempting to claim ownership of the child’s objects, their own objects, and objects belonging to the other experimenter (see Fig. 2). These data were entered into a 2(Population: TD, ASD) × 3(Owner: Child, Experimenter, “Thief”) mixed ANOVA.

Significant main effects of Owner, *F*(2, 70) = 8.51, MSE = 0.22, *p* < .001, η_p_^2^ = .20, and Population, *F*(1, 35) = 4.70, MSE = 0.77, *p* = .037, η_p_^2^ = .12, were qualified by a significant Population × Owner interaction, *F*(2, 70) = 4.43, MSE = 0.22, *p* = .015, η_p_^2^ = .11. When the thief attempted to claim the child’s object, TD children (*M* = 0.79) were significantly more likely to protest than children with ASD (*M* = 0.17; *p* = .021). When the thief attempted to claim the other experimenter’s object, TD children (*M* = 0.63) tended to protest more often than children with ASD (*M* = 0.17; *p* = .063). Protest frequencies did not significantly differ between TD children (*M* = 0.05) and children with ASD (*M* = 0.06) when the thief claimed ownership of their own object (*p* = .97). For children with ASD, protest frequencies did not significantly differ between the three owners. By contrast, TD children were significantly more likely to protest when then the thief attempted to claim ownership of items made by the participant (*p* = .007) and the experimenter (*p* = .017) than the thief’s own items.

**Fig. 3:**
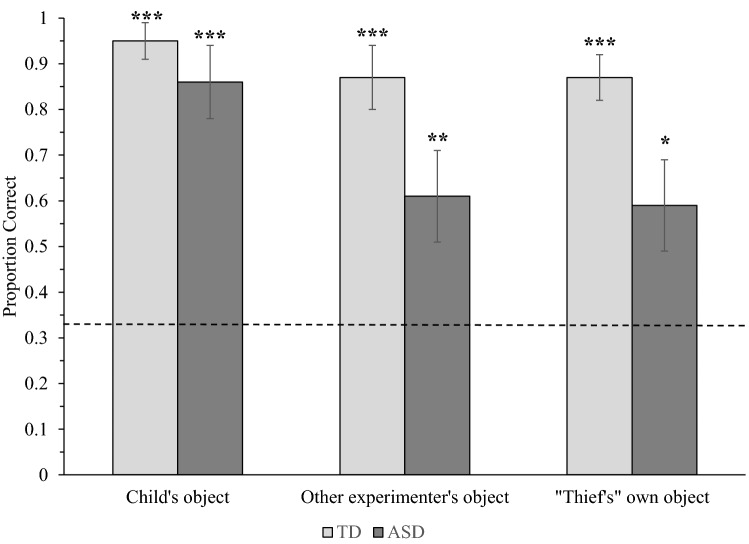
Mean accuracy on ownership questions for typically developing (TD) children and children with autism spectrum disorder (ASD) in the ownership rights task. Error bars show ± 1 SE. Stars above columns indicate where performance was significantly more accurate than expected by chance, indicated by the dotted line (*p < .05; **p < .01; ***p < .001)

Children were also scored out of six corresponding to the number of ownership questions answered correctly (three per trial; see Fig. 3). These data were entered into a 2(Population: TD, ASD) × 3(Owner: Child, Experimenter, “Thief”) mixed ANOVA.

The results revealed a significant main effect of Owner, *F*(2, 70) = 6.80, MSE = 0.21, *p* = .002, η_p_^2^ = .16. Children identified objects belonging to them (*M* = 1.81) with significantly greater accuracy than objects belonging to the thief (*M* = 1.45, *p* = .011) and marginally greater accuracy than objects belonging to the other experimenter (*M* = 1.48, *p* = .056). The main effect of Population was also significant, *F*(1, 35) = 6.22, MSE = 0.78, *p* = .017, η_p_^2^ = .15, indicating that TD children (*M* = 1.79) responded to ownership questions with significantly greater accuracy than children with ASD (*M* = 1.37).

## Discussion

This study investigated how ASD affects children’s ability to identify ownership from linguistic cues and their understanding of ownership rights. The ownership identification task revealed that children with ASD responded less accurately than TD children when ownership relationships were stated and probed using possessive pronouns. Children with ASD were also less accurate at identifying the belongings of third parties. The results of the ownership rights task showed that children with ASD were less likely to protest in defence of their own and others’ ownership rights than TD children. Both populations reliably identified each party as the owner of the items they created, but children with ASD were less accurate at assigning ownership on this basis than TD children. Together, these findings suggest that children with ASD are less accurate at tracking owner-object relationships when ownership status is not explicitly stated with reference to proper nouns and they may have reduced sensitivity to breaches in ownership rights.

As predicted, children with ASD were significantly less accurate at identifying owner-object relationships when they were required to comprehend possessive pronouns (e.g. mine, yours). This finding suggests that previously documented difficulties using and comprehending personal pronouns (e.g. I, you; e.g. Tager-Flusberg 1994) may impact on children’s understanding of ownership. It is likely that difficulties associated with personal and possessive pronouns share the same underlying cause. Differences in representing the self in relation to others (Overweg et al. 2018) and executive functioning (Dale and Crain-Thoreson 1993) may impair deictic shifting—the ability to dynamically remap pronouns to different referents depending on who is speaking. For example, a lunchbox belonging to Jack could be referenced using “mine”, “yours”, or “his”—words that can also refer to different objects belonging to other owners. By contrast, children with ASD demonstrated broadly comparable accuracy to TD controls when identifying belongings via proper nouns. Proper nouns do not require deictic shifting and have fixed relationships with referents (e.g. a lunchbox belonging to Jack is always and only “Jack’s lunchbox”). This one-to-one mapping reduces referential ambiguity and facilitates cross-situational associative learning, which is a strength in ASD (Foti et al. 2015; Hartley et al. 2020b; Roser et al. 2015).

Children with ASD were also less accurate than TD controls at tracking relationships between other people and their property. This finding contrasts with the results of Hartley and Fisher (2018a), which showed that children with ASD could identify the property of others with ceiling-level accuracy. The samples in the two studies were similar in terms of chronological age and language comprehension, so it seems unlikely that the disparity is due to demographic variability. However, there are two important methodological differences that could be influential. Firstly, in Hartley and Fisher (2018a), the three objects in each set were visually distinct, clearly contrasting on both shape and colour (e.g. a whistle shaped like a bird vs. an eraser shaped like a zebra vs. a multi-coloured slinky). In our ownership identification task, the three objects in each set belonged to the same category (e.g. three differently-coloured lunch boxes) and thus shared similarities in global shape. It is possible that the ability of children with ASD to identify owner-object relationships benefits from greater perceptual discriminability in addition to the use of fixed reference terms. Secondly, in Hartley and Fisher (2018a), children with ASD may have paid closer attention to the objects that did not belong to them because they were offered the opportunity to trade for them. Given that children with ASD often traded their randomly assigned item for a preferred alternative, it is likely that they studied each of the objects and this encoding may have facilitated their subsequent owner-object matching. Conversely, in our task, children with ASD may have been less attentive to the other-owned objects because they lacked instrumental motivation to study them closely.

Also in accord with our predictions, children with ASD were less likely to protest in defence of their own and others’ ownership rights than TD children. While neither group protested when the thief attempted to claim ownership of their items, only the TD group demonstrated awareness of ownership rights by blocking attempts to claim non-owned property. The results of our TD sample (*M* age = 3.88 years) were broadly consistent with those generated by Kanngiesser and Hood’s (2014a) sample of 3-year-olds (*M* age = 3.50 years) in their similar task. Our TD participants were approximately 10% less likely to defend their own ownership rights (39.5% vs. ~ 50%), but approximately 10% more likely to defend the ownership rights of a third party (31.5% vs. ~ 20%). The absence of protests in the ASD group cannot be attributed to a failure to map owner-object relationships; like the TD group, children with ASD reliably assigned objects to owners based on investment of creative labour at above-chance rates (see Kanngiesser and Hood 2014b; Kanngiesser et al. 2010). One possibility is that the ASD group were aware that owners’ rights were being violated, but they were unmotivated or unwilling to intervene. However, casting doubt on this hypothesis, nearly two-thirds of the ASD sample demonstrated willingness to correct the thief’s erroneous behaviour in the warm-up game and it is surprising that they did not at least defend their own rights (as TD 2-year-olds do; Rossano et al. 2011).

Alternatively, it may be that the ASD group did not believe that ownership conferred the right to control access to their created objects, so the thief’s actions were not considered to be a transgression. Previous research has demonstrated that, in stark contrast to TD controls, children with ASD do not display robust effects associated with ownership. For example, children with ASD do not prefer *their* objects merely due to ownership (Hartley and Fisher 2018a) and they do not over-value authentic items belonging to famous owners (Hartley et al. 2020a). These effects have been linked to reduced concern for abstract relationships between people and property. According to the ‘extended-self hypothesis’, establishing ownership forges a connection between a person and an item, transforming the item into a physical marker of their identity (Belk 1988; Hood et al. 2016). In turn, an abstract trace of the self transfers to the object (Argo et al. 2008). This mentalistic connection to property explains why self-owned possessions are more memorable, desirable, and judged to be more valuable than similar non-owned items (Cunningham et al. 2013; Gelman et al. 2012, 2015; Kahneman et al. 1991). However, early differences in developing a psychological sense of self (Lind 2010), coupled with difficulties engaging in social learning through interactions (APA 2013), may affect the psychological influence of ownership and the development of associated concepts. Consequently, autistic development may be characterised by reduced awareness of ownership rights and decreased sensitivity when these are breached.

Importantly, differences in ownership understanding could have implications for children’s ability to navigate the social world. The lack of protests in defence of ownership rights aligns with previous evidence that children with ASD are more accepting of a partner’s unfair behaviour in resource-sharing games (e.g. Hartley and Fisher 2018b; Sally and Hill 2006). These traits may increase children’s vulnerability to bullies exploiting their lower concern for property ownership. For example, children with ASD could potentially be at increased risk of property theft if they do not understand their ownership rights or actively defend them. Moreover, lack of awareness that others have ownership rights could lead children with ASD to unwittingly interact with non-owned property without invitation. Failure to conform with ownership norms may result in children with ASD being perceived as ‘different’ by their peers (Humphrey and Lewis 2008; van Roekel et al. 2010), hindering their formation and maintenance of positive interpersonal relationships (Bauminger and Kasari 2000; Chamberlain et al. 2007). The findings from our owner identification task suggest that caregivers and teachers ought to be mindful of the language they use when communicating about owner-object relationships with children with ASD, favouring the use of proper nouns (e.g. “Did Nina take John’s pencil?”) rather than pronouns (e.g. “Did you take his pencil?”) to increase the likelihood of comprehension. It is also important to note that our experimental tasks were highly-structured and ownership-relevant information was extremely salient. Thus, it is possible that between-population differences would be more prominent if participants were required to independently infer connections between people and property through observation of naturalistic behaviour.

We recommend that future research expands the exploration of how ASD affects children’s understanding of ownership. Awareness of how ownership influences behaviour and feelings towards objects can help children predict and understand others’ actions and emotions in social situations (Pesowski and Friedman 2015, 2018). It has been hypothesised that these aspects of ownership cognition may be related to children’s development of Theory of Mind (Rochat 2011). While some recent evidence suggests that Theory of Mind does not support TD children’s identification of owner-object relationships (McDermott and Noles 2018; Rochat et al. 2014), deficits in mentalising associated with ASD could potentially contribute to differences in understanding ownership rights, and other as-yet-unstudied facets of ownership cognition, that play important roles in social interaction. It is also currently unknown how ASD impacts children’s identification of ownership via non-linguistic cues, such as possession and social stereotypes (Friedman and Neary 2008; Malcolm et al. 2014), or understanding of ownership transfer (e.g. buying and gift giving; Blake and Harris 2009). Additionally, our finding that children with ASD show reduced sensitivity to ownership rights could have implications for their understanding of bodily rights. In typical development, understanding of ownership rights and bodily rights are related (Van de Vondervoort et al. 2017; Van de Vondervoort and Friedman 2015)—it has been hypothesised that they both depend on the principle that people have autonomy over things that are theirs and that the former may stem from the latter (Neary and Friedman 2014). Thus, if children with ASD do not understand their own and others’ rights over objects, they may also display differences in their awareness of rights associated with bodily contact, potentially increasing their risk of victimisation (see McEachern 2012; Mandell et al. 2005). Further research investigating these aspects of ownership understanding in ASD could play a valuable role in highlighting psychological mechanisms that contribute to children’s behavioural difficulties associated with communication and interaction, and potentially identify targets for intervention.

Of course, we must address the limitations of this study. Firstly, it is possible that the observed between-population differences were related to general limitations in cognitive functioning in the ASD sample or differences in experience (the ASD group were significantly older than the TD controls). We acknowledge that including a sample of children with delayed intellectual development matched to children with ASD on non-verbal intelligence and chronological age would have eliminated this issue. Secondly, it is possible that children with ASD did not protest against the thief’s behaviour in the ownership rights task because they were an adult, and thus perceived to be an authority figure. However, this limitation may be mitigated by the fact that most children with ASD were willing to correct their erroneous behaviour in the warm-up game and the protest rates of our TD participants were similar to those reported by Kanngiesser and Hood (2014a) who employed a puppet as the thief. In addition, previous studies have reported that children with ASD are increasingly accepting of behaviour that breaches social norms when playing games with a puppet (e.g. Hartley and Fisher 2018b). Nevertheless, it would be valuable for future research to analyse how children with ASD interact with owned and non-owned property in naturalistic interactions with parents and siblings (O’Brien et al. 2020; Ross 2013). Finally, due to the novelty of this research, we recognise that further studies are required to replicate our findings, address our limitations, and provide further insight into relationship between differences in ownership understanding and behavioural features of ASD.

In summary, the present study reports the first evidence that children with ASD are less accurate than TD children when mapping relationships between people and their property. In particular, children with ASD have difficulty identifying ownership based on possessive pronouns and they may struggle to keep track of others’ belongings. We also showed, for the first time, that children with ASD are less likely to defend their own and others’ ownership rights. We hypothesise that these results may be attributed to differences in representing the self and propose that ASD may be characterised by reduced concern for ownership and atypical development of associated concepts, such as ownership rights. Our findings inform broader understanding of social-cognitive differences associated with autism and highlight the possibility that children with ASD could face problems in social situations that require understanding of ownership rules.
